# New Appliances and Things Medical

**Published:** 1905-04-15

**Authors:** 


### NEW APPLIANCES AND THINGS MEDICAL.

[We shall be glad to receive, at our Office, 28 & 29 Southampton Street, Strand, London, W.C., from the manufacturers, specimens of all new preparations and appliances which may be brought out from time to time.]

### THE PATENT CHRISTIA TISSUE.

J. Christy and Co., Old Swan Lane, E.

This tissue is intended to supersede oiled silk for surgical and other purposes where a light, pliant, and waterproof fabric is required. We are glad to bring it to the notice of our readers, as it is a novelty which has much to recommend it. It is especially suitable in connection with the application of fomentations. For domestic use it possesses the advantage of cheapness. This permits of its being freely discarded after use, whilst the more expensive and durable oil silks are not infrequently kept for use again in the future from motives of economy.

